# Flavonoid compounds of buah merah (*Pandanus conoideus* Lamk) as a potent SARS-CoV-2 main protease inhibitor: in silico approach

**DOI:** 10.1186/s43094-021-00309-0

**Published:** 2021-08-10

**Authors:** Abd. Kakhar Umar

**Affiliations:** Department of Pharmaceutics and Pharmaceutical Technology, Faculty of Pharmacy, Universitas Padjadjaran, Jatinangor, 45363 Indonesia

**Keywords:** Quercetin 3′-glucoside, Quercetin 3-O-glucose, Taxifolin 3-O-α-arabinopyranose, COVID-19, Anti-SARS-CoV-2 agents

## Abstract

**Background:**

COVID19 is a global pandemic that threatens all nations. As there is no effective antiviral drug for COVID19, we examined the potency of natural ingredients against the SARS-CoV-2 main protease (PDB ID 6YNQ). Buah merah is a typical fruit from Papua, Indonesia, which is known to contain high levels of carotenoids and flavonoids. The contents have been proven to be effective as antiparasitic and anti-HIV. An in silico approach to 16 metabolites of buah merah (*Pandanus conoideus* Lamk) was carried out using AutoDock Vina. Furthermore, the study of the dynamics of ligand–protein interactions was carried out using CABS Flex 2.0 server to determine the test ligand and receptor complexes' stability. ADMET prediction was also carried out to study the pharmacokinetic profile of potential antiviral candidates.

**Result:**

The docking results showed that 3 of the 16 buah merah metabolites were potent inhibitors against the SARS-CoV-2 main protease. The flavonoid compounds are quercetin 3′-glucoside, quercetin 3-O-glucose, and taxifolin 3-O-α-arabinopyranose with a binding affinity of − 9.7, − 9.3, and − 8.8, respectively, with stable ligand–protein complex. ADMET study shows that the three compounds are easily dissolved, easily absorbed orally and topically, have a high unbound fraction, low toxicity, and non-irritant.

**Conclusion:**

We conclude that quercetin 3′-glucoside, quercetin 3-O-glucose, and taxifolin 3-O-α-arabinopyranose can be used and improved as potential anti-SARS-CoV-2 agents in further study.

## Background

The 2019 coronavirus disease (COVID19) has had a significant impact on all countries in the world. Based on a WHO report accessed on Apr 12, 2021, there were 4 million new cases in 1 week, with an increase of 11% compared to last week, with over 71.000 new deaths reported [1]. The virus that causes COVID19 is severe acute respiratory syndrome coronavirus 2 (SARS-CoV-2), which belongs to the β-coronavirus group. Inhibiting virus replication is one method of reducing the severity of infection. The major proteins known to be responsible for the life cycle of viruses have been reported. This protein plays a role in the maturation process of viral proteins to become a target in developing new antivirals [2–4].

Several secondary metabolites from plants have been reported to have good inhibitory activity against the SARS-CoV-2 main protease. The terpenoid and triterpenoid compounds are the strongest inhibitor [2, 3]. Previous studies reported that rutin had the best inhibitory effect, even compared to the FDA-approved COVID19 antiviral (Remdesivir) [2, 5]. This terpenoid is known to bind stably to the SARS-CoV-2 main protease receptor (PDB ID 6YNQ). Since the 6YNQ protein has never been identified to have mutations, it has been an appealing focus in developing antivirals using the in silico approach [2].

Buah merah is a typical Indonesian plant that has been widely used by Indonesian people, especially Papua, as medicine or daily food. Buah merah contains high levels of carotenoid and flavonoid metabolites. Buah merah (*Pandanus conoideus* Lamk) has been reported to have antioxidant, antitumor, immunomodulatory, antiparasitic, and anti-HIV effects [6–9]. As buah merah has good antiparasitic and anti-HIV potential, suggesting potential activity against the SARS-CoV-2 main protease [3]. Therefore, this study was conducted to see whether the flavonoid and carotenoid content of buah merah inhibited SARS-CoV-2 main protease compared to rutin and remdesivir. Molecular dynamics were studied to see how the relationship and stability of the ligand–protein complexes. ADMET prediction was also performed to assess the pharmacokinetic profile of potential drug candidates.

## Methods

### Ligand preparation

*Pandanus conoideus* Lamk was reported to contain carotenoids and flavonoids. (The list can be seen in Table 1.) [10, 11] The ligand structure was drawn using ChemDraw Pro 12.0. The ligand structure was then trimmed using ChemDraw's “clean structure” feature, and their energy was minimized (MM2) using Chem3D. The ligand structure was then saved into PDB format. The ligands were optimized again using AutoDockTools 1.5.6 (ADT) (TheScripps Research Institute, the USA) to add Gasteiger charges, set rotatable bonds, and TORSDOF. All ligands were then saved into PDBQT format. The structures of rutin, astragalin, trifolin, and remdesivir were obtained from the PubChem database. The reference ligand was then optimized just like the previous test ligand.

**Table 1:** Binding affinity of carotenoid and flavonoid compounds of *Pandanus conoideus* Lamk, reference flavonoids, and resemdivir against SARS-CoV-2 main protease (6YNQ)

Compound	Binding affinity
*Carotenoids*	
5,6-diepicapsokarpoxanthin	− 7.5
Capsorubin	− 6.8
Capsanthin 5,6-epoxide	− 7.8
Capsanthin 3,6-epoxide	− 7.1
Capsanthin	− 7.1
Cryptocapsin	− 7.5
β-cryptoxanthin 5,6-epoxide	− 7.7
Cryptoxanthin	− 7.0
*Flavonoids*	
4′,6,6′,8-tetrahydroxy-3-methoxy-flavon	− 7.7
3,4′,5-trihydroxy-7,3′-dimethoxy flavon	− 7.5
Taxifolin 3-O-α-arabinopyranose	− 8.8
Quercetin 3-O-glucose	− 9.3
Quercetin 3-methyl-ether	− 7.6
Quercetin	− 7.8
Taxifolin	− 8.4
Quercetin 3′-glucoside	− 9.7
Rutin	− 8.4
Astragalin	− 8.2
Remdesivir	− 7.5
Trifolin	− 7.8

### Protein preparation

SARS-CoV-2 main protease was obtained from the protein data bank (PDB) on http://www.rscb.org/pdb/ with the protein code 6YNQ. Native ligand and protein were separated using the Discovery Studio 2021 Client (DS) (BIOVIA, San Diego, CA, the USA). The protein was optimized using ADT to remove water, regulate the charges (Kollman charges), and add polar hydrogen. The protein was then stored in PDBQT format. The grid position was arranged based on the active site attached by the native ligand. The XYZ axis of the protein was set to 5,870, − 0.017, 19,615. The grid dimension was set to 40 × 40 × 40 magnification with a spacing of 0.375 Å.

### Molecular docking

The docking process was carried out using AutoDock Vina. The operating system used was Windows 10 Home Single Language 64 bit with AMD Ryzen 5 3500U, Radeon Vega Mobile Gfx 2.10 GHz, and RAM of 8 GB. The energy range was set to 4 and exhaustiveness to 8. The output file was made in PDBQT format used for visualization of docking results. 2D and 3D visualization was done using DS.

### Molecular dynamics study

The ligand–protein interaction dynamics study was carried out to determine the most active amino acid residues at the binding site of the SARS-CoV-2 main protease. The output file from the docking process produces 9 ligand–protein interaction models for each ligand. All the active amino acid residues bind to the ligand, and their number of occurrences has been observed and recorded.

The protein's stable structure was studied using the CABS Flex 2.0 server, which is based on coarse-grained simulations of protein motion [12]. The number of cycles and trajectory frames was set to 50, with a global weight of 1.0 and a temperature of 1.4. The distance restraints generator was set to default values. This test aims to see whether the ligand–protein interaction remains stable during attachment [2].

### ADMET prediction

The pharmacokinetics profile of the selected potential ligands was studied using pkCSM ADMET to determine the sterol compound's quality and safety. The SMILES string for each ligand is obtained from a PDB ligand file converted to SMI format using DS.

## Results

### Structural features of SARS-CoV-2 main protease and binding affinity of *Pandanus conoideus* Lamk compounds

**Fig. 1:**
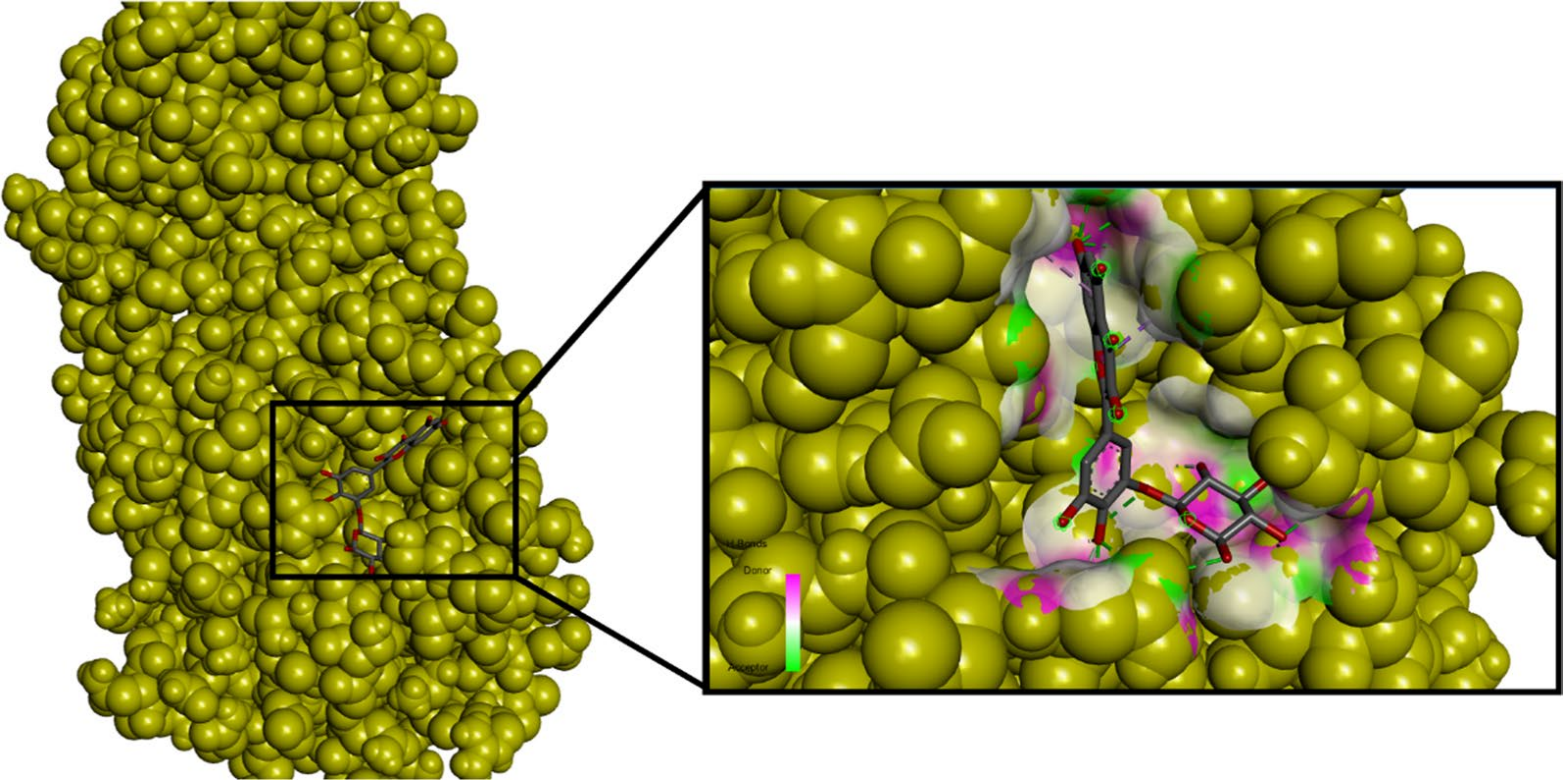
Binding pocket of SARS-CoV-2 main protease (6YNQ) with quercetin 3′-glucoside

The total active amino acid residues at the binding site of the SARS-CoV-2 main protease were 23, including Asn142, Arg188, Cys44, Cys145, Gln189, Gln192, Glu166, Gly143, His43, His164, Leu141, Leu167, Met49, Met165, Phe140, Pro168, Ser46, Ser144, Thr24, Thr45, Thr45, and Thr190. The protein used (6YNQ) binding site appears to have approximately the same amino acid residue as 6LU7 [2, 3]. Most of the amino acid residues in the 6YNQ binding pocket are hydrogen donors or acceptors (see Fig. 1).

**Fig. 2:**
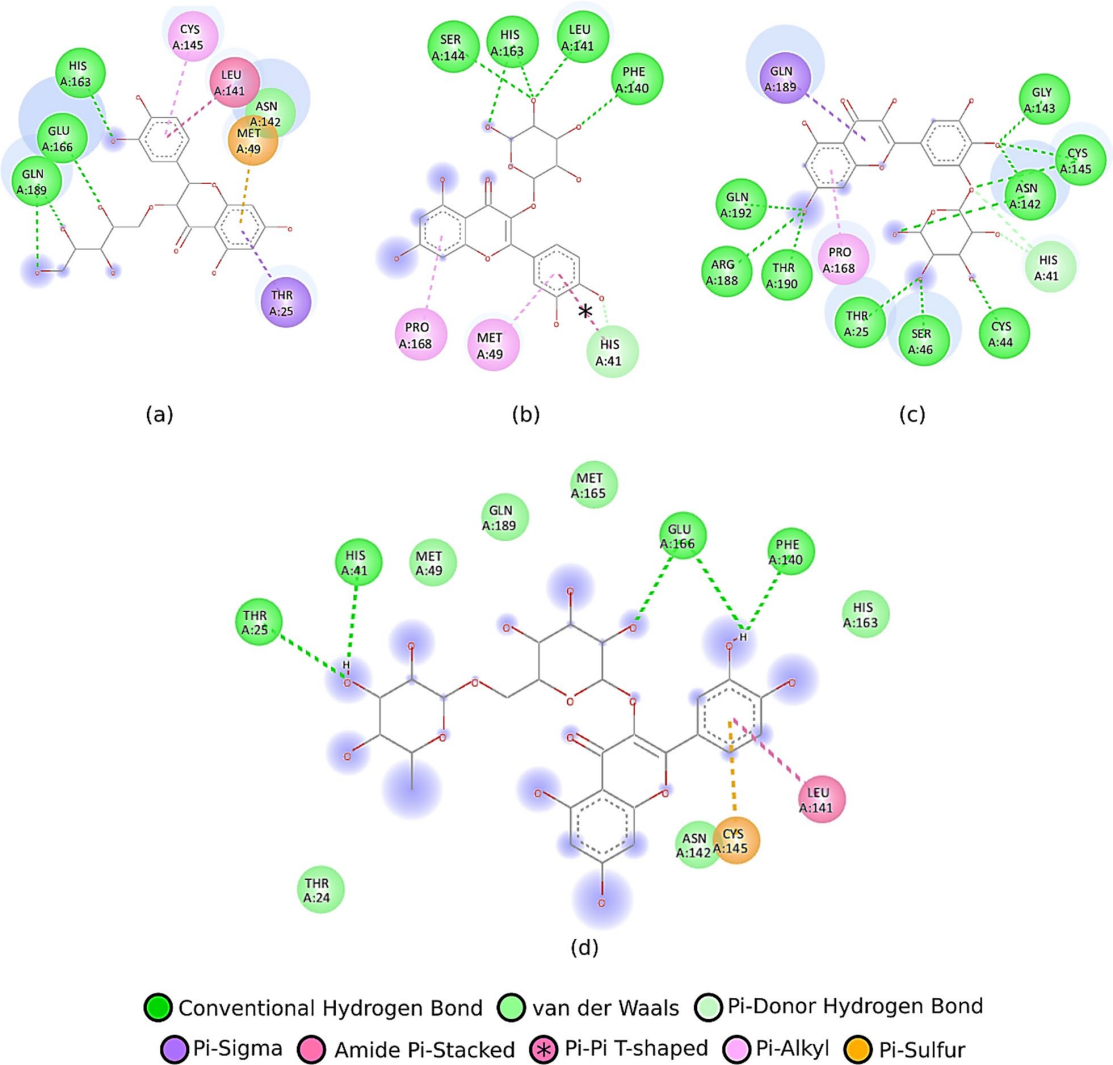
Ligand–protein interactions of **a** taxifolin 3-O-α-arabinopyranose, **b** quercetin 3-O-glucose, **c** quercetin 3′-glucoside, and **d** rutin

**Fig. 3:**
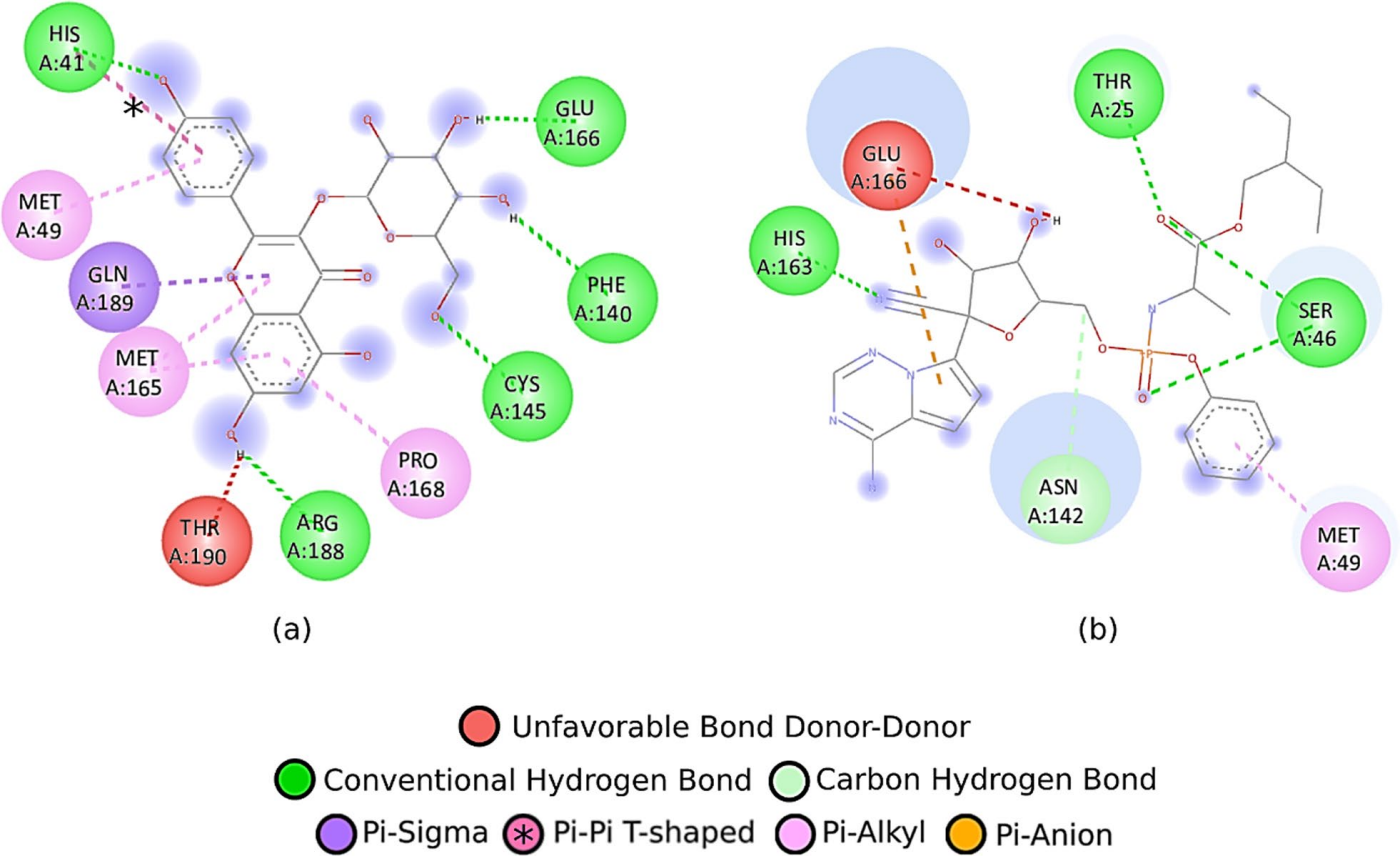
Ligand–protein interactions of **a** astragalin and **b** remdesivir

Based on the docking results, the flavonoid component of *Pandanus conoideus* Lamk had the strongest binding affinity, while the carotenoid component only ranged from − 6.8 to − 7.8. In Table 1, it can be seen that quercetin 3′-glucoside has the highest binding affinity, which is − 9.7, then followed by quercetin 3-O-glucose (− 9.3) and taxifolin 3-O-α-arabinopyranose (− 8.8). This value was significant than the reference flavonoid and antiviral. Rutin only gets a binding affinity score of − 8.4, then followed by astragalin (− 8.2), trifolin (− 8.7), and remdesivir (− 7.5). Previously, rutin, astragalin, trifolin, and remdesivir have been investigated through an in silico approach as potential inhibitors of the SARS-CoV-2 main protease at the same protein code tested in this study (6YNQ) [2]. Compared to the rutin structure, quercetin 3′-glucoside has a structure that fits perfectly with the binding pocket of the SARS-CoV-2 main protease (see Fig. 1). Quercetin 3′-glucoside also binds to more amino acid residues than the reference ligands. Hydrogen bonds formed on quercetin 3-O-glucose are two times more than rutin with double hydrogen bonds on the Asn142 and Cys145 residues (see Fig. 2). Asn142 and Cys145 are known to be the catalytic active site residue of the SARS-CoV-2 main protease, so that forming bonds to these residues will produce strong inhibition [2, 12]. Apart from Asn142 and Cys145, the test ligands with the strongest binding affinity have similar interactions with several amino acid residues, including Gln189, Glu166, and His41. The reference ligands (astragalin and remdesivir) have a lower binding affinity than rutin. This result is consistent with the previous study [2]. It has also been shown that astragalin and remdesivir form unfavorable donor bonds in the residues of Thr190 and Glu166 (see Fig. 3).

### Dynamics of ligand–protein interactions and the stable form of SARS-CoV-2 main protease

**Fig. 4:**
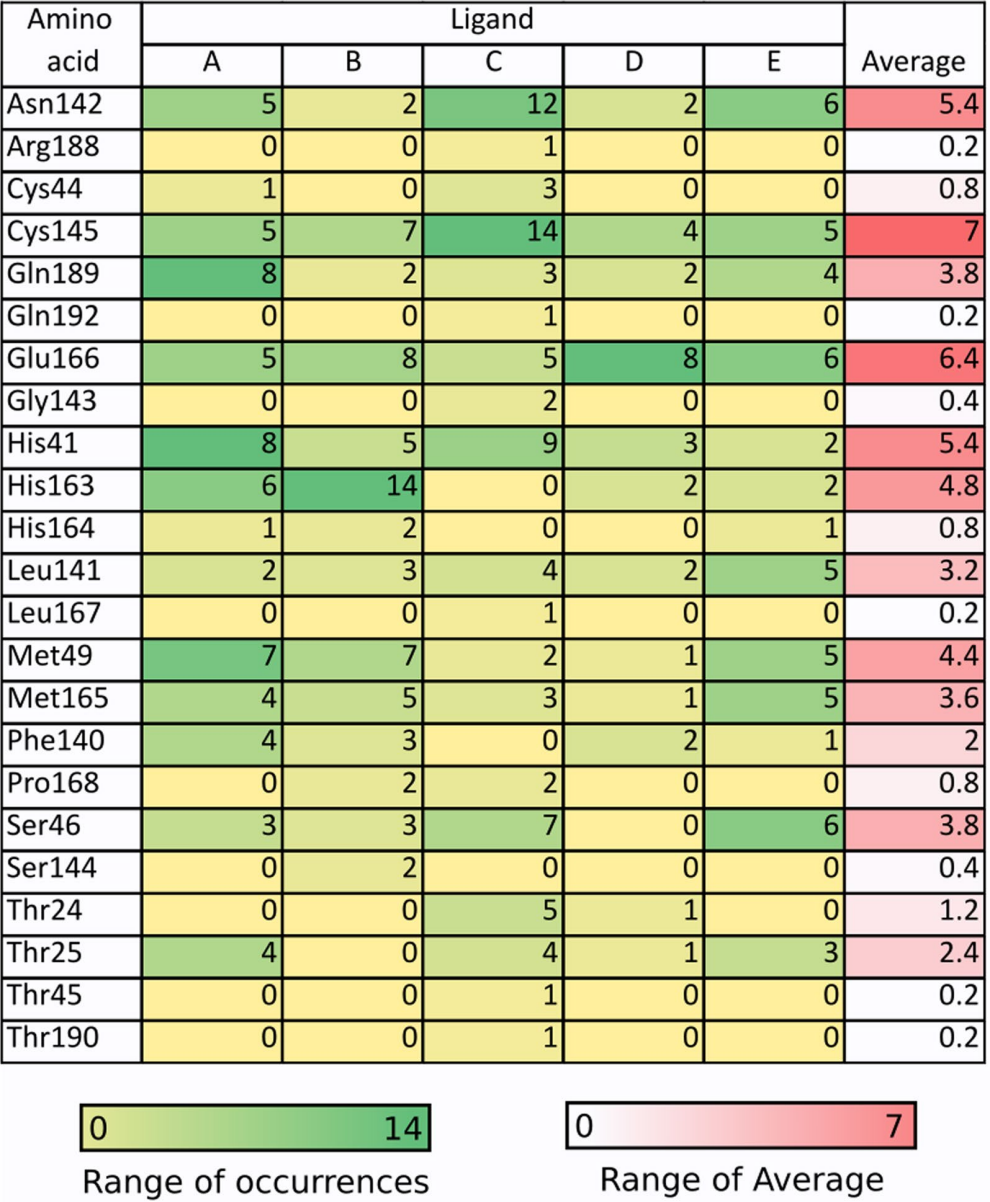
Dynamics of ligand–protein interactions of A taxifolin 3-O-α-arabinopyranose, B quercetin 3-O-glucose, C quercetin 3′-glucoside, D rutin, and E resemdivir. *Note*: The value is based on the number of occurrences in each interaction mode. Double bonds count as two

The dynamics of ligand–protein interactions were studied to determine which amino acid residues interact with the ligands most frequently and rank the most active amino acid residues in all ligands. This test was only performed on a potent compound obtained from previous molecular docking studies, including quercetin 3′-glucoside (− 9.7), quercetin 3-O-glucose (− 9.3), dan taxifolin 3-O-α-arabinopyranose (− 8.8) as well as with reference flavonoid (rutin, − 8.4) and FDA approved antiviral (remdesivir, − 7.5). In Fig. 4, it can be seen that taxifolin 3-O-α-arabinopyranose tends to interact more with Gln189, His41, and Met49 with occurrence scores of 8, 8, and 7, respectively. Quercetin 3-O-glucose tends to form bonds with His163, Glu166, Cys145, and Met49 with occurrence scores of 14, 8, 7, and 7, respectively. Meanwhile, quercetin 3′-glucoside is likely to interact with Cys145, Asn142, His41, and Ser46 with occurrence scores of 14, 12, 9, and 7, respectively. Only Glu166, Ser46, and Cys145 are the most active residues in their interactions with the receptor on the reference ligands.

**Fig. 5:**
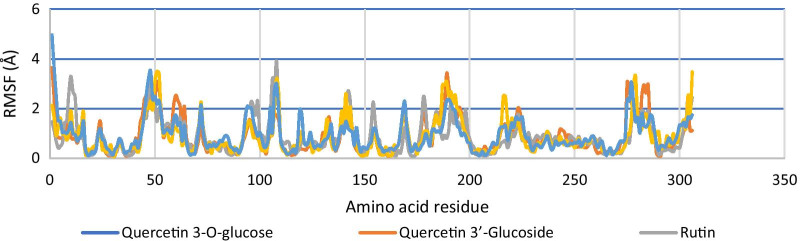
Root mean square fluctuations in protein structures in response to specific substrates

**Fig. 6:**
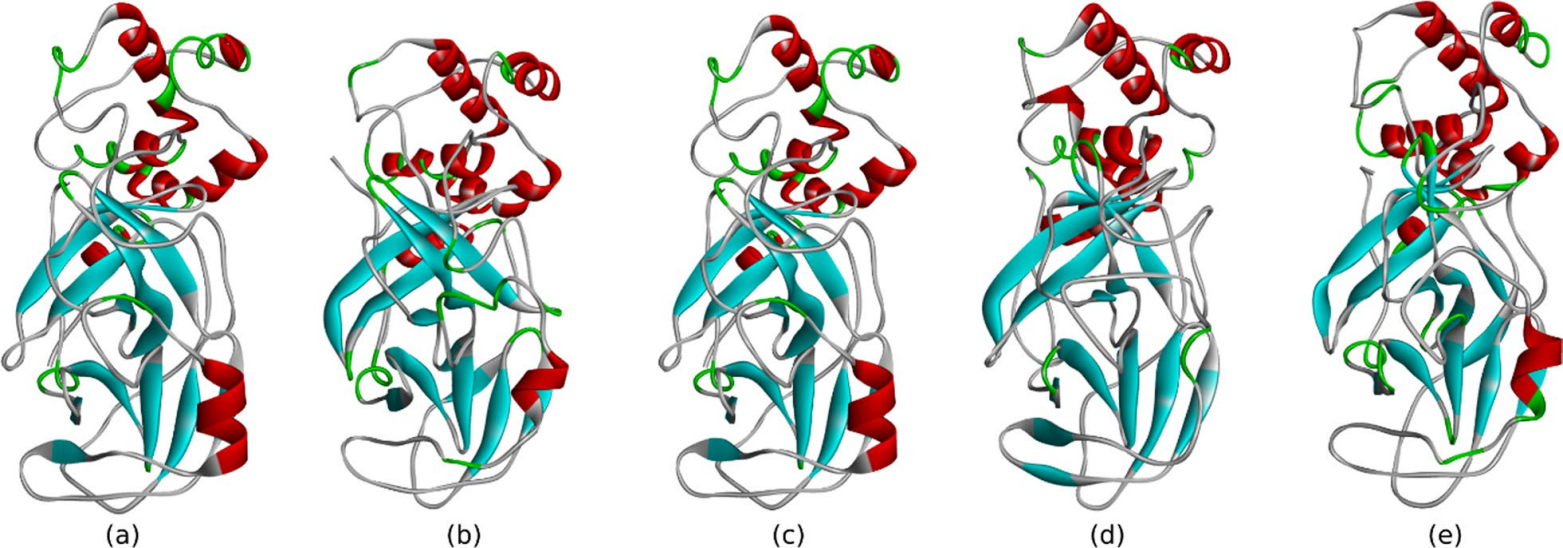
Stable protein structures of **a** taxifolin 3-O-α-arabinopyranose, **b** quercetin 3-O-glucose, **c** quercetin 3′-glucoside, **d** rutin, and **e** resemdivir, generated following molecular dynamics simulations

When interacting with SARS-CoV-2 main protease, all potent test ligands and reference ligands showed more than 90% amino acid residue yielding RMSD < 2 Å. The fluctuation of the root means square can be seen in Fig. 5. From these results, the interaction of each test ligand and reference ligand with protein forms a stable complex. The stable structure of each of the ligand–protein complexes can be seen in Fig. 6.

### ADMET prediction

The ADMET prediction results showed that the three potent test ligands showed a good pharmacokinetic profile. ADMET prediction data can be seen in Table 2.

**Table 2:** ADMET prediction of three potent SARS-CoV-2 main protease (6YNQ) inhibitors

Model name	Ligand		
	A	B	C
*Absorption*			
Water solubility (log mol/L)	− 3.078	− 2.941	− 2.898
Caco2 permeability (log Papp in 10^−6^ cm/s)	− 0.637	− 0.773	− 0.68
Intestinal absorption (human) (% Absorbed)	51.047	30.619	35.708
Skin permeability (log Kp)	− 2.735	− 2.735	− 2.735
P-glycoprotein substrate	Yes	Yes	Yes
P-glycoprotein I inhibitor	No	No	No
P-glycoprotein II inhibitor	No	No	No
*Distribution*			
VDss (human) (log L/kg)	0.624	0.061	0.332
Fraction unbound (human)	0.224	0.207	0.22
BBB permeability (log BB)	− 1.835	− 2.139	− 2.408
CNS permeability (log PS)	− 4.382	− 4.777	− 4.748
*Metabolism*			
CYP2D6 substrate	No	No	No
CYP3A4 substrate	No	No	No
CYP1A2 inhibitior	No	No	No
CYP2C19 inhibitior	No	No	No
CYP2C9 inhibitior	No	No	No
CYP2D6 inhibitior	No	No	No
CYP3A4 inhibitior	No	No	No
*Excretion*			
Total clearance (log ml/min/kg)	− 0.007	0.568	0.437
Renal OCT2 substrate	No	No	No
*Toxicity*			
Max. tolerated dose (human) (log mg/kg/day)	0.933	0.754	0.618
hERG I inhibitor	No	No	No
hERG II inhibitor	No	No	No
Oral rat acute toxicity (LD50) (mol/kg)	2.548	2.71	2.581
Oral rat chronic toxicity (LOAEL) (log mg/KgBB/day)	3.707	4.024	5.229
Hepatotoxicity	No	No	No
Skin Sensitization	No	No	No

(A) Taxifolin 3-O-α-arabinopyranose, (B) quercetin 3-O-glucose, (C) quercetin 3′-glucoside

## Discussion

The SARS-CoV-2 main protease does have many hydrogen donors and acceptors in its binding pocket. This can be seen in the interaction of quercetin 3′-glucoside with the receptor, where nine hydrogen bonds are formed, likewise, for other ligands where the hydrogen bond is dominant. This can be utilized for more optimal ligand development by targeting the hydrogen bonds in the amino acids Asn142 and Cys145, which are crucial amino acids [13–15]. Among the potent test ligands, quercetin 3′-glucoside most frequently interacts with Cys145 on all modes, acting as a catalytic active site residue. The ranking of amino acid residues' occurrence showed that Cys145, Glu166, Asn142, and His41 were the residues that played the most significant role in interacting with ligands.

Drugs can be classified based on their solubility. Drugs with a LogS value >  − 2 show high solubility, the range − 2 to − 4 is slightly soluble, and <  − 4 is insoluble. Based on the results of the ADMET prediction study, it can be seen that all potent ligands have good solubility. Value of HIA > 30% and LogKp <  − 2.5 demonstrated that all potent ligands have good oral absorption and skin penetration. All potent ligands are also not included as substrates or inhibitors of P-glycoprotein I/II. This shows that P-glycoprotein does not assist the absorption of all potent ligands. All potent ligands also have no contraindication with other drugs whose absorption is assisted by P-glycoprotein [16].

All potent ligands' distribution is also excellent where the log Vdss value is >  − 0.15, and the free fraction in plasma is > 20%. The higher the logVdss value, the more drug fraction distributed to the tissue than in plasma. The more free fraction, the more efficient and the smaller the dose of drug needed. All the test ligands also showed low blood barrier penetration (logBB <  − 1 and logPS <  − 3), so that it can be said that the ligands would not directly affect the central nervous system. In terms of metabolism, all potent ligands are not substrates or inhibitors of cytochrome P450, so it can be said that all test ligands are not metabolized by cytochrome P450 and do not interfere with the metabolism of other drugs [16]. Quercetin 3′-glucoside, quercetin 3-O-glucose, and taxifolin 3-O-α- arabinopyranose have a total clearance of 0.437, 0.568, and − 0.007, respectively, and are not a substrate of OCT2.

The maximum human tolerable dose of quercetin 3′-glucoside, quercetin 3-O-glucose, and taxifolin 3-O-α-arabinopyranose is 4.15, 5.67, and 8.57 mg/KgBB/day, respectively. All test ligands are not hERG I and II inhibitors and therefore do not potentially cause fatal ventricular arrhythmia. The oral rat acute and chronic toxicity of each potent ligand can be seen in Table 2. All potent ligands are not hepatotoxic and non-irritant.

## Conclusion

The terpenoid compounds of buah merah (*Pandanus conoideus* Lamk) have potent inhibitory activity against the SARS-CoV-2 main protease. Quercetin 3′-glucoside, quercetin 3-O-glucose, and taxifolin 3-O-α-arabinopyranose are potent inhibitors with a binding affinity − 9.7, − 9.3, and − 8.8, respectively. The three compounds that have an excellent pharmacokinetic profile are non-hepatotoxic and non-irritant. Based on this in silico study, we conclude that quercetin 3′-glucoside, quercetin 3-O-glucose, and taxifolin 3-O-α-arabinopyranose can be used and improved as potential anti-SARS-CoV-2 agents in further study.

## Data Availability

All data and materials are available upon request.

## Abbreviations

COVID-19: Coronavirus disease 2019
SARS-CoV-2: Severe acute respiratory syndrome coronavirus-2
PDB: Protein data bank
ADMET: Absorption, distribution, metabolism, excretion, and toxicity
HIV: Human immunodeficiency virus
ADT: AutoDockTools 1.5.6
DS: Discovery studio 2021 client
RMSD: Root mean square distance
RMSF: Root mean square fluctuation
